# Crosstalk between Platelets and SARS-CoV-2: Implications in Thrombo-Inflammatory Complications in COVID-19

**DOI:** 10.3390/ijms241814133

**Published:** 2023-09-15

**Authors:** Junyi Zhao, Xiafan Xu, Yifei Gao, Yijing Yu, Conglei Li

**Affiliations:** School of Medicine, The Chinese University of Hong Kong, Shenzhen 518172, China; junyizhao@link.cuhk.edu.cn (J.Z.); xiafanxu@link.cuhk.edu.cn (X.X.); yifeigao2@link.cuhk.edu.cn (Y.G.)

**Keywords:** platelet, thrombosis, SARS-CoV-2, COVID-19

## Abstract

The SARS-CoV-2 virus, causing the devastating COVID-19 pandemic, has been reported to affect platelets and cause increased thrombotic events, hinting at the possible bidirectional interactions between platelets and the virus. In this review, we discuss the potential mechanisms underlying the increased thrombotic events as well as altered platelet count and activity in COVID-19. Inspired by existing knowledge on platelet–pathogen interactions, we propose several potential antiviral strategies that platelets might undertake to combat SARS-CoV-2, including their abilities to internalize the virus, release bioactive molecules to interfere with viral infection, and modulate the functions of immune cells. Moreover, we discuss current and potential platelet-targeted therapeutic strategies in controlling COVID-19, including antiplatelet drugs, anticoagulants, and inflammation-targeting treatments. These strategies have shown promise in clinical settings to alleviate the severity of thrombo-inflammatory complications and reduce the mortality rate among COVID-19 patients. In conclusion, an in-depth understanding of platelet–SARS-CoV-2 interactions may uncover novel mechanisms underlying severe COVID-19 complications and could provide new therapeutic avenues for managing this disease.

## 1. Introduction

The coronavirus disease 2019 (COVID-19) caused by severe acute respiratory syndrome coronavirus 2 (SARS-CoV-2) was recognized by the World Health Organization as a pandemic from March 2020 to May 2023, and it has claimed nearly 7 million lives since December 2019 (https://www.who.int/emergencies/diseases/novel-coronavirus-2019, accessed on 2 July 2023). The economic burden caused by COVID-19 is also tremendous. Most COVID-19 patients exhibit mild to moderate symptoms; however, at the beginning of pandemic, around 15% of patients developed severe pneumonia, and approximately 5% progress to acute respiratory distress syndrome or/and multiple organ failure [1,2]. Age (>50 years) and pre-existing medical conditions (e.g., cancers, cardiovascular diseases, and diabetes) are among the risk factors for severe symptoms in COVID-19 [3]. In addition to life supportive care and antiviral therapies treating severe patients, vaccination is the most effective strategy to prevent COVID-19-related mortality [4,5].

To gain entry into host cells, SARS-CoV-2 binds to the angiotensin-converting enzyme 2 (ACE2) receptor, which is widely expressed in many cells, such as lung and intestinal epithelial cells, monocytes, dendritic cells, and macrophages [6]. SARS-CoV-2 can activate both innate and adaptive immune responses, resulting in uncontrolled inflammatory innate responses and defective adaptive immune responses [3]. Indeed, a cytokine storm had been observed in severely ill COVID-19 patients, and most of these patients exhibited markedly increased serum levels of proinflammatory cytokines, such as interleukin (IL)-6, tumor necrosis factor alpha (TNF-α), GM-CSF, and IL-1β [1,3,6,7,8]. Another key feature of severe COVID-19 patients was lymphopenia, since reduced numbers of CD4+ and CD8+ T cells, B cells, and NK cells were commonly observed in those patients [1,2,9].

In spite of causing lymphopenia, SARS-CoV-2 is capable of eliciting IgG and IgA antibody responses, as well as CD4^+^ (predominantly Th1) and CD8^+^ T cell responses in the majority of infected individuals with mild symptoms [10,11]. It was found that anti-SARS-CoV-2 IgM and IgA antibodies were detectable in the early days of post-symptom onset (median time of appearance: day 5), while anti-SARS-CoV-2 IgG antibodies appeared a little later (around day 14 post-symptom onset). Since anti-SARS-CoV-2 antibodies could neutralize the viruses and attenuate the pathogenesis of COVID-19, the neutralizing antibodies have been used to treat severe COVID-19 patients [12,13,14,15]. In addition to antibody production, SARS-CoV-2 infection could induce polyclonal CD4^+^ and CD8^+^ T cell responses targeting over 2000 unique epitopes and rapid type I interferon responses. Early T cell responses have been associated with mild COVID-19 symptoms [5,16,17]. Notably, severe or fatal COVID-19 are characterized by fewer CD8^+^ T cells and profound disruption of germinal center responses against SARS-CoV-2 [15,18,19,20]. In contrast, high levels of innate immune cells, such as monocytes and neutrophils, are observed in the lungs of patients with fatal COVID-19 [21,22,23]. Thus, antibody responses are critical to prevent SARS-CoV-2 infection, while T cell responses may play important roles in reducing the disease severity and controlling viral infections [5,15,24]. Furthermore, while the emergence of new SARS-CoV-2 variants weakens the neutralizing antibody responses, most CD4^+^ and CD8^+^ T cell responses remain preserved [5,25].

Surprisingly, severe COVID-19 patients exhibit a profound hypercoagulable state, and thrombotic complications are common [26]. Excessive clotting has been observed in around 25–30% of severely ill COVID-19 patients [27,28]. Klok and colleagues evaluated the frequency of venous thromboembolism and arterial thrombotic events in 184 severe COVID-19 patients in the Netherlands and found that around 27% of patients developed venous thromboembolism, while around 4% of patients exhibited arterial thrombotic events [27]. None of these COVID-19 patients developed diffuse intravascular coagulation [27]. In another study, Cui and colleagues determined the incidence of venous thromboembolism in 81 severe COVID-19 patients in China and observed that 25% of patients developed venous thromboembolism [28]. Consistent with these findings, postmortem studies revealed disseminated microthrombi in the lung of COVID-19 patients [29]. Interestingly, race and ethnicity have a major impact on the thrombotic risk in COVID-19 among different populations. In addition, thrombocytopenia has also been observed in around 5–36% of COVID-19 patients [1,9]. The meta-analyses have indicated that platelet amount is strongly associated with the severity and mortality of COVID-19, and reduced platelet amount can act as a biomarker for a poor prognosis [30,31].

Our previous review article has discussed the important antimicrobial effect of platelets and put a question mark on the role platelets play in SARS-CoV-2 infection [32]. Are platelets friends or foes in COVID-19? Do platelets’ functions alternate along with the staging of SARS-CoV-2 infection? These important questions remained to be addressed. In this review, we firstly summarize the functions of platelets, retrospect thrombotic events, and platelet alterations in COVID-19 cases, and then we propose potential antiviral strategies utilized by platelets in a SARS-CoV-2 infection, followed by a discussion on targeting platelets in treating COVID-19.

## 2. Roles of Platelets in Physiology and Pathobiology

Platelets, as products of megakaryocytes, are anucleate cells that circulate in the human blood and are vitally involved in hemostasis and thrombosis [33,34,35]. Traditionally, it is believed that platelets are generated in the bone marrow of mammalians; however, in accordance with the recent murine studies, platelets could also derive from megakaryocytes residing in the lung [34]. Long known for their importance in hemostasis and thrombosis, recent research has highlighted the versatility of platelets, as evidenced by their involvement in many other physiological processes such as inflammation, antimicrobial and antiviral immunity, and tumorigenesis [36,37]. In addition, platelets also act as a central contributor in a range of pathological conditions from mild to severe, including thrombocytosis and thrombocytopenia. Many clinical observations have confirmed that COVID-19 patients could develop thrombotic pathologies like thrombocytopenia and thrombosis due to the SARS-CoV-2-induced platelet activation [38,39,40]. 

### 2.1. Platelets in Hemostasis and Thrombosis

Hemostasis is a multiple-step process that results in the arrest of bleeding, starting from the adhesion, activation to the aggregation of platelets at the damaged sites of blood vessels [41]. Platelets possess two major kinds of glycoprotein receptors on their surface: the GPIbα complex and GPIIb/IIIa complex (also termed integrin αIIbβ3). GPIbα, via binding to the von Willebrand factor (vWF), is required for platelets to adhere to the injured vessel wall, while integrin αIIbβ3, via interacting with fibrinogen and other ligands, is required for platelet aggregation [42,43,44,45]. Thrombin generation, which is amplified by activated platelets, sparks a downstream coagulation cascade, resulting in a fibrin clot that blocks blood leakage at the sites of vascular injury [46]. Pathological platelet activation will cause the inappropriate formation of blood clots, also called thrombi, in blood vessels, initiating thrombosis [47,48]. In addition to platelet activation, aberrant activation of the intrinsic or extrinsic pathway of coagulation can lead to the formation of intravascular clots, which contribute to both arterial and venous thrombosis [49]. Thrombosis is considered as the primary cause of arterial diseases accounting for stroke and myocardial infraction, as well as venous thromboembolic disorders associated with severe morbidity and mortality [50].

### 2.2. Platelets in Innate and Adaptive Immunity

Owing to their expression of many kinds of inflammation-related molecules, platelets contribute to both innate and adaptive immunity via various mechanisms. In host innate immunity, platelets can generate antimicrobial factors, such as defensins or kinocidins, and enhance neutrophil-dependent phagocytosis to eliminate pathogens like viruses, bacteria, and parasites [51,52]. Platelets express several important pathogen-recognition receptors (PRRs), including Toll-like receptors (TLRs) 1–10 and Nod-like receptor 2 [53,54,55,56]. Functional chemokine receptors CCR3, CCR4, and CXCR4 are also expressed on the surface of platelets [57]. Additionally, platelets can release both pro-inflammatory molecules (e.g., serotonin and CD40) and anti-inflammatory molecules and cytokines (e.g., TGF-β) from granules, thereby regulating inflammatory responses [53,58]. Platelets also shape adaptive immune system in the host. Platelets express CD40L, a key molecule in the antibody class switch process and CD8^+^ cytotoxic T cell function [59]. TGF-β, secreted by platelets, can not only suppress the CD8^+^ T cell function in a tumor and enhance regulatory T cell response but can also play a crucial role in antibody isotype switching to IgA, which participates in modulating gut microbiota homeostasis [60,61,62].

In addition, platelets recruit leukocytes towards thrombi to exacerbate inflammation. Accumulating evidence implies that platelets are instrumental for recruiting leukocytes to sites of inflammation. After being activated, platelets express P-selectin and secrete multiple kinds of pro-inflammatory molecules, including platelet factor 4 (PF4), platelet-activating factor, and neutrophil-activating peptide, guiding leukocytes to migrate from blood circulation towards where the thrombi form. These leukocytes then get activated and initiate subsequent events through the interaction between their integrins and the adhesion molecules distributed on the thrombi. For instance, the complete activation of leukocyte integrin starts from the ligation between platelet P-selectin and P-selectin glycoprotein ligand 1 (PSGL-1) on leukocytes, allowing leukocytes to tether and roll on the thrombi via the interactions between integrins and other platelet ligands (e.g., fibrinogen, GPIbα, and ICAM-2). Activated leukocyte integrins interact with fibrinogen/fibrin in thrombi, leading to a series of pro-inflammatory events such as leukocyte migration, extravasation, degranulation, and superoxide production [63]. Furthermore, the neutrophil extracellular traps (NETs), produced by activated neutrophils at the low shear pocket area of thrombi, also play a role in inflammation [64]. Another study has demonstrated that platelets accumulated at the sites of inflamed vascular surface can attract 20-fold more leukocytes compared to the same sites without platelet aggregates [65]. Based on the above findings, we conclude that thrombi provide a reactive platform for platelets to recruit leukocytes and promote their migration across the vessels. Thus, it is possible that platelet-mediated thrombotic activities at the SARS-CoV-2 invasion site of lung pre-alveolar vessels may aggravate the inflammatory state through recruiting leukocytes from the circulation [66].

### 2.3. Platelets in Microbial Infections

Due to the ability of releasing pro-inflammatory molecules, and the fact that reduced platelet counts reflect the high susceptibility and poor resistance of host-to-pathogen infections, platelets are capable of providing protection to the host against microbial infections using various means [67,68]. Platelets act as pathogen sensors in the early stage of invasion and also get recruited to breach sites [54,69]. As effectors in the anti-pathogen process, platelets not only exert direct encapsulating or killing effects on pathogens but also indirectly eliminate pathogens with the help of immune cells [58]. In addition to innate immunity, platelets could also mediate adaptive immune response responding to pathogenic infection. For instance, in a mouse model, platelets are found to transfer Listeria monocytogenes from the blood circulation to CD8a^+^ dendritic cells in the spleen and promote the clonal expansion of CD8^+^ cytotoxic T cells [70]. Although platelets are involved in bacterial and parasite infections [67,71,72,73,74,75,76,77], in this review, we mainly focus on the role of platelets in viral infections. 

Platelets have been demonstrated to impact the pathogenesis of a few DNA virus infections, such as cytomegalovirus (CMV), vaccinia, or herpes simplex virus type 1 (HSV-1); however, the mechanisms are still not well understood [54,78,79]. It had been shown that TLR2 expressed on the surface of the platelet could bind to human CMV, which led to the release of vascular endothelial growth factors (VEGF), IL-1β, and CD154. Although enhanced aggregation or adhesion was not observed in human CMV-activated platelets, they can recruit neutrophils and form heterotypic aggregates. Besides the platelet–neutrophil aggregation, it was also found that the interactions between human CMV-activated platelets and other immune cells were elevated, including dendritic cells, B lymphocytes, T lymphocytes, and monocytes. Sottnek and colleagues had reported that lethal intravascular coagulation mediated with platelets was observed at 24 h post vaccinia virus infection [80]. Another study had stated that HSV-1 infection could cause damage to human umbilical vein endothelial cells, resulting in increased thrombin production and higher cellular binding capacity to platelets [81].

Compared to DNA viral infections, platelets could respond to RNA viral infections in more diverse ways. Platelets can directly internalize RNA viruses including dengue, coxsackievirus B, HIV, hepatitis C, and encephalomyocarditis viruses [82,83,84,85]. Previous studies reported that the degree of thrombocytopenia varies with the staging of RNA viral infections. Koupenova et al. had observed platelet–neutrophil aggregates, which decreased platelet count in encephalomyocarditis-virus-infected mice. Additionally, in the same study, platelets were found to be required for the host survival via a platelet–TLR7-interaction-dependent manner using the TLR7-knockout mice model [82]. Dengue virus infection could cause severe hemorrhagic fever, as well as thrombocytopenia in the host. Based on the study with the dengue-infected rhesus macaque model, platelets were found to interact with various immune cells, such as neutrophils and monocytes. Platelets internalizing the dengue virus were subsequently engulfed by monocytes [86]. 

## 3. Thrombotic Events and Platelet Alteration in COVID-19 

Platelets are increasingly viewed as crucial mediators of inflammation and other immune disorders [87,88]. Based on previous autopsy studies, high platelet reactivity was linked with dispersed thrombosis in multiple organs, which suggested that SARS-CoV-2-mediated platelet activation, in turn, contributed to the pathophysiology of COVID-19 [21]. In cases of COVID-19 pneumonia, researchers have observed higher levels of platelet-granulocyte and platelet–monocyte aggregates [89]. In the following sections, how platelets are altered and how platelet–leukocyte interaction leads to thrombotic events in COVID-19 will be discussed.

### 3.1. Thrombotic Events in COVID-19 

Thrombotic events are commonly identified in both clinical diagnoses and autopsies of COVID-19 patients. D-dimer, which is derived from the degradation of fibrin in fibrinolysis, can act as a biomarker for thrombotic events [90]. It was confirmed that high D-dimer concentration in circulation was associated with thrombosis in COVID-19 infections [91]. Wu and colleagues had reported markedly higher serum D-dimer levels in non-survived COVID-19 patients compared to survivors, indicating a significant risk of thrombosis in COVID-19 infections [92].

Venous thromboembolism, which includes deep vein thrombosis (DVT) and pulmonary embolism, is a common type of thrombotic event [93]. In a meta-analysis involving 3342 patients, the pooled incidence rate of DVT and pulmonary embolism were 14.8% and 16.5%, respectively [94]. For those mild patients who did not develop pulmonary embolism, microthrombosis was also prevalent. Integrated with a histological study, pulmonary microthrombi were reported in 57% of COVID-19 patients, and this frequency is similar to that in SARS-infected patients (58%)—and much higher than that in influenza-infected patients (24% in H1N1)—indicating intense thrombotic effects of SARS-CoV-2 [95]. DVT and pulmonary embolism were present even more commonly in critically ill COVID-19 patients. Among 34 patients hospitalized in intensive care units (ICU) in France, up to 79% of patients were diagnosed with DVT via ultrasonogram [96]. In an autopsy study, 7 of 12 cases were found to exhibit DVT, and massive pulmonary embolism was confirmed as the cause of death in four cases [97]. Though severe illness and prolonged immobility (such as ICU hospitalization) were thought to contribute to DVT, several clinical studies had revealed that COVID-19 patients showed a much higher DVT incidence rate than non-COVID-19 patients admitted to ICU with acute respiratory distress syndrome or influenza, which strengthened the notion that COVID-19 promoted thrombotic events [27,98,99]. 

Besides venous thrombotic events, arterial thrombosis was also reported in COVID-19 patients. Since higher blood flow rate hinders the formation of arterial thrombus, the incidence rate of arterial thrombus in COVID-19 patients was indeed much lower than that of venous thrombus. Nevertheless, arterial thrombosis was more prevalent in COVID-19 patients compared with other diseases. Klok et al. investigated 198 COVID-19 patients admitted to ICU, and all received at least standard doses thromboprophylaxis. Arterial thrombosis was found in 3.7% of patients [27]. A similar study reported 2.8% of arterial thrombosis in over 400 COVID-19 patients [91].

### 3.2. Platelet Count and Volume Change in COVID-19 Patients

The characteristics and functions of platelets are altered in many infectious diseases. Specifically in coronaviridae infections, thrombocytopenia was found in half of patients infected with severe acute respiratory syndrome coronavirus (SARS-CoV), and also half of patients exhibited prolonged activated partial thromboplastin time, indicative of blood clotting defects [100]. For individuals with mild symptoms, thrombocytopenia was usually a self-limiting condition, with platelet counts typically returning to normal within two weeks. Interestingly, half of patients developed thrombocytosis two weeks later after the onset of SARS-CoV infection [101]. Similarly for the Middle East respiratory syndrome coronavirus (MERS-CoV), one study tracking 47 MERS-CoV-infected patients showed that one third of affected patients developed thrombocytopenia [102]. Hwang et al. also observed the relatively lower platelet count in MERS-CoV-infected patients compared to healthy controls [103].

Recent studies demonstrated a significant reduction in platelet count in COVID-19 patients, indicating a potential role for platelets in the pathophysiology of this disease. In an integrated meta-analysis exploring the association between severe COVID-19 and low platelet count, 4582 individuals from 15 studies were included. The results revealed that 23% of the subjects developed thrombocytopenia, with the ratio significantly higher in severe patients (42.9%) compared to non-severe patients (16.9%) [30]. It has also been observed that the change in platelet count over the course of the disease would be a factor affecting the prognosis of COVID-19, with a common reduction in platelet number at the onset of this disease. For those who survived the disease, platelet count gradually increased overall, while for non-survivors, the platelet count declined in the later stages of the disease course [104].

In addition to changes in platelet count, COVID-19 patients exhibited higher frequencies of immature platelets, as measured with an immature platelet fraction and immature platelet count, compared to non-COVID-19-hospitalized patients [105]. Moreover, platelet volume had been found to correlate with the disease severity of COVID-19. Specifically, mean platelet volume had been reported to be higher in non-survivors of COVID-19. In addition, mean platelet volume was significantly higher in COVID-19 patients compared to non-COVID-19 patients [106,107]. Elevated mean platelet volume in the blood circulation may indicate a larger proportion of young platelets, which could be a result of the body’s response to thrombocytopenia [108]. 

Currently, the underlying mechanisms of reduced platelet count remain unclear. Previous research on other related coronaviruses and recent studies about the pathophysiology of SARS-CoV-2 infection suggested several potential reasons. These include that (1) platelet production might be interrupted in COVID-19 infections. Many coronaviruses, including SARS and human coronavirus 299E, had been reported with capability of recognizing aminopeptidase N (CD13 in human) and infecting cells that express CD13 [109,110]. CD13 is commonly expressed on the cell surface of megakaryocytic cells and platelets [111]. Consequently, SARS-CoV-2 may disturb hematopoiesis to reduce platelet formation in patients. (2) The evidence that lungs are a major site of platelet biogenesis indicates lung injury in COVID-19, and thrombocytopenia can also be well correlated [112]. (3) Damaged lung tissue and pulmonary endothelium cells may lead to platelet activation and aggregation, resulting in thrombi in lungs [113]. The platelet consumption due to excessive clot formation could lead to thrombocytopenia. Apparently, this speculation was strengthened with the observation of elevated immature platelet fraction in COVID-19 patients, implying a replenishment of platelets in circulation after thrombi formation.

### 3.3. Platelet Activation in COVID-19

#### 3.3.1. Platelets Are Sensitized in COVID-19 Patients

**Figure 1:**
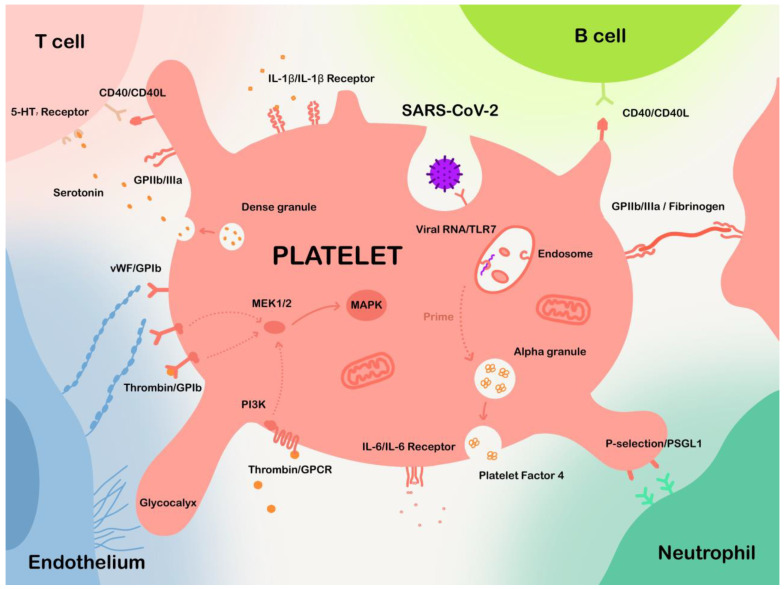
The interactions between platelets and different cells in COVID-19. Platelets are capable of binding to multiple types of cells, including endothelial cells, neutrophils, T cells, B cells, and also many other cells. Platelets may also engulf SARS-CoV-2. Internalized viral RNA may be recognized using Toll-like receptors in endosome, leading to invocation of multiple anti-viral strategies.

Thrombotic events in COVID-19 patients may result from multiple mechanisms, including lung-injury-induced hypoxia, endothelial dysfunction, excessive inflammation, stasis, and also platelet activation [114,115,116,117]. Indeed, platelet activation is commonly found in infectious diseases, leading to many studies researching on and revealing the feature of platelet activation in COVID-19. Degranulation is an important approach for platelets to perform their functions. Two most common types of granules, α granules and dense granules, fuse with the platelet membrane, and their contents get released into plasma upon platelet activation [74]. PF4 and serotonin are contained in α granules and dense granules, respectively. To compare platelet granule release capacity between COVID-19 patients and healthy subjects, Zaid et al. tested PF4 and serotonin levels in plasma and platelets, respectively. They found that, for COVID-19 patients, PF4 and serotonin levels were significantly higher in plasma than platelets compared to the healthy subjects, indicating platelets were activated in COVID-19 patients and released more granules than healthy people (Figure 1) [118]. Meanwhile, PKCδ, a key regulator in platelet activation, was found to be more phosphorylated in platelets of COVID-19 patients compared to the healthy group [118]. 

#### 3.3.2. Mechanisms of Platelet Activation in COVID-19 

Several extrinsic mechanisms of platelet activation will be discussed, including inflammation, endothelial dysfunction, and direct invasion of SARS-CoV-2 into platelets.

##### Inflammation-Induced Platelet Activation

Similar to many other infectious diseases, inflammation-induced platelet activation was also pronounced in COVID-19 patients. Besides the tissue injury directly induced by the SARS-CoV-2 virus, uncontrolled inflammation, or so-called cytokine storms, are also known to contribute to the pathogenesis of COVID-19 [119]. Indeed, IL-6, IL-1β, and tumor necrosis factor alpha (TNF-α) were found to be significantly elevated in COVID-19 patients [6]. Summarized by Iba et al., circulating IL-6 level in COVID-19 patients was comparably high to that in other diseases exhibiting cytokine storms, including cytokine release syndrome after chimeric antigen receptor T cell therapy and hemophagocytic lymphohistiocytosis [120]. Earlier studies had found that circulating IL-6, IL-1β, and TNF-α were capable of sensitizing platelets to activation [121,122,123]. These inflammatory cytokines stimulated platelets to express more tissue factors [124] and release extracellular vesicles [118], resulting in more platelet activation and thrombotic events. 

##### Endothelial Dysfunction Influences Platelet Activation and Aggregation 

In addition to cytokine stimulation, endothelial dysfunction also contributes to platelet activation as an extrinsic factor. This is because the interaction between platelets and endothelial cells is crucial to platelet functions. Blood endothelial cells synthesize von Willebrand factors (vWF) in their endoplasmic reticulum [125]. vWF is a large multimeric glycoprotein that aids platelets to adhere to sites of vascular injury. Normally, vWF is cleaved by enzyme A Disintegrin and Metalloprotease with Thrombospondin motifs-13 (ADAMTS13); however, the involvement of inflammatory cytokines such as TNF-α can devitalize ADAMTS13 and trigger the release of vWF, leading to platelet activation and aggregation (Figure 1) [126]. Endothelial cells also express endothelial glycocalyx on the vascular lining, which normally inhibits platelet adhesion. However, in the presence of inflammation, endothelial glycocalyx sheds more from the vascular lining, resulting in platelet adhesion (Figure 1) [127]. There are also other signal molecules involved in the platelet–endothelium interaction, including nitric oxide and prostacyclin. In the presence of inflammation, the biosynthesis of nitric oxide and prostaglandin I2 is interrupted, leading to diminishment of antiplatelet function of endothelium [66]. 

Besides inflammation, direct invasion is another pathway for SARS-CoV-2 to induce endothelial dysfunction. As mentioned above, ACE2 is expressed in the endothelial cells of many distinct organs, including the lung and heart [128]. Autopsies verified that SARS-CoV-2 could infect endothelial cells and cause endotheliitis [129]. Although the specific infection mechanisms of SARS-CoV-2 remained unclear, the changes in redox homeostasis in affected cells were presented as a common feature of respiratory viral infections and shared similar consequences with inflammation on endothelial cells [130,131]. 

##### SARS-CoV-2 May Invade Platelets to Affect Their Function

Some studies also suggested that SARS-CoV-2 may directly invade platelets to affect their function. Autopsies reported the presence of SARS-CoV-2 viral particles in both megakaryocytes and platelets [132]. Interestingly, while Zhang et al. reported the expression of ACE2 on the surface of platelets [40], other studies failed to repeat this observation [118,132,133]. The contradiction questioned whether SARS-CoV-2 interacted with platelets through ACE2. Consequently, CD147 was proposed as an alternative receptor for SARS-CoV-2 on platelets [134]. Unfortunately, this novel pathway was challenged with no obvious binding against SARS-CoV-2 spike protein [135]. Actually, among several proposed receptors, which included ACE2, CD209, CD299, C-type lectin domain family 10 member A (CLEC10A), CLEC4G, CD147, and asialoglycoprotein receptor 1 [136], Barrett et al. revealed that only CD147 was found among platelet transcripts via RNA-seq, as well as on megakaryocytes via flow cytometry [132]. However, more in-depth investigations are required to explain the mechanism of SARS-CoV-2 entry into platelets. Nevertheless, SARS-CoV-2 was verified to be engulfed by platelets or megakaryocytes, and internalized SARS-CoV-2 was capable of altering metabolic pathways in platelets, such as oxidative phosphorylation and glycolysis (Figure 1) [130]. 

#### 3.3.3. Platelet Transcriptome Alteration 

The platelet activation process relies not only on the extrinsic stimulations but also on the intrinsic transcriptome changes. Several studies profiling platelet transcriptome alterations had revealed some pathways enriched in COVID-19. However, our current understanding of this research area is still limited. Manne et al. observed that the mitogen-activated protein kinase (MAPK) pathway was up-regulated in platelets from COVID-19 patients [133]. The molecular mechanisms through which SARS-CoV-2 alter platelet transcriptome remain in mists. Platelets contain pre-mRNA and mRNA derived from megakaryocytes [137]. Consequently, SARS-CoV-2 may affect gene expression in megakaryocytes, therefore altering platelet transcriptome. However, more in-depth investigations are required to uncover the mechanisms involved in this process.

#### 3.3.4. Signaling Effects on Platelet Activation

The process of platelet activation is induced by the invocation of a series of signaling cascades. Previous studies have indicated their potent stimulation to platelet activation and aggregation [138], among which MAPK cascades are verified to be up-regulated in platelets from COVID-19 patients, as mentioned above [133]. It has been known that MAPK signaling cascade activates platelet cytosolic phospholipase A2 (cPLA_2_) to promote thromboxane production, thereby increasing thrombus generation. Manne and colleagues also reported the increase in cPLA_2_ phosphorylation in platelets from COVID-19 patients [139]. When it comes to upstream signaling events of MAPK cascade, several pathways have been shown to invoke MAPK in platelets. For example, GPIb-IX signaling, induced by vWF or thrombin, can lead to the downstream activation of phosphoinositide 3-kinase (PI3K) and finally MAPK pathways (Figure 1) [138]. Meanwhile, G-protein-coupled receptors on the platelets, which can also be induced by thrombin, activate the PI3K-MAPK cascade (Figure 1) [138]. Two well-studied MAPK proteins, ERK1/2 and p38, are reported to function in human platelet activation [140]. ERK1/2 and p38 are capable of regulating cPLA_2_ [141,142], as phosphorylation increment of cPLA_2_ is reported by Manne et al. [133]. P38 can also activate MAPK-activated protein kinase 2 [143]. These cascades finally result in an activation of GPIIb/IIIa, leading to platelet activation and aggregation. Additionally, p38 can phosphorylate purinergic receptor P2Y_12_ [144], resulting in a reduction in intracellular cyclic adenosine-3′,5′-monophosphate levels and therefore stabilizing platelet aggregation [145].

In addition to MAPK cascades, many other signaling pathways have been demonstrated with previous studies to function in platelet activation. Phospholipase C, which may be activated by NADPH oxidase and thrombin receptors [146,147,148], can result in Ca^2+^ release from dense tubular system into the cytosol, leading to calcium elevation, which is involved in plenty of platelet-activation-related signaling [138]. LIM kinase 1, which shares a similar up-stream regulatory network with MAPK, can mediate thromboxane A2 synthesis and subsequently promote thrombosis [149]. However, none of these pathways are verified in COVID-19 patients. Thus, more studies are needed to validate their involvement in SARS-CoV-2 infections.

## 4. Potential Antiviral Effects of Platelets on SARS-CoV-2

As mentioned above, extensive studies revealed the altered platelet count and activation status as well as enhanced thrombotic events in COVID-19 patients, and several mechanisms underlying SARS-CoV-2-mediated platelet activation have been proposed. However, according to our previous discussion, platelets also exert effects on multiple pathogens through direct or indirect means to eliminate them. Similar strategies might be utilized by activated platelets to combat SARS-CoV-2 infection. Based on accumulative knowledge of how platelets modulate bacterial, parasitic, and viral infections, we would put forward the following potential antiviral mechanisms that platelets might undertake to control SARS-CoV-2 infection.

### 4.1. Internalizing SARS-CoV-2 

The most common antiviral response of platelets relies on their abilities to sequester viruses via internalization, subsequently preventing viruses from pervading the host circulation system. Previous studies have reported that platelets internalize single-strand RNA viruses, and the RNA genome will be recognized by and bound to TLR7 in the lysosome. Activated TLR7 may cause the release of α granules, leading to the surface expression of CD40L and P-selectin that can mediate platelet–leukocyte interaction. 

**Figure 2:**
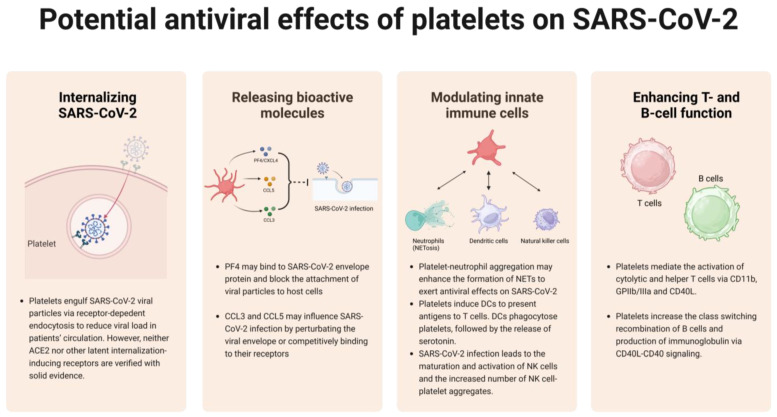
Platelets may respond to SARS-CoV-2 invasion via several approaches. Though unclear with recognizing mechanisms, platelets can engulf SARS-CoV-2 to reduce viral load. Platelets can also release cytokines to antagonize viral invasion. Moreover, platelets help with modulating and enhancing innate and adaptive immune cells to boost immune functions.

In the context of SARS-CoV-2 infection, it has been found that SARS-CoV-2 mRNA can be detected in platelets isolated from COVID-19 patients [133]. SARS-CoV-2 internalization is likely to influence metabolism of platelets therefore induces thrombotic events as mentioned above, and on the other hand, it is a potential approach to reduce viral load in patients’ circulation (Figure 2). Platelets may internalize SARS-CoV-2 particles via ACE2-dependent endocytosis [133]. However, whether platelets express ACE2 is still controversial. Several studies have claimed that SARS-CoV-2-induced platelet activation via ACE2-spike protein interaction supported the claim that ACE2 is expressed in platelets [40,150]. In contrast, others have shown that both mRNA and protein of ACE2 could not be detected in platelets [135,136]. It seems that platelets may be able to engulf SARS-CoV-2, but since neither ACE2 nor other latent internalization-inducing receptors [118,133] are verified with solid evidence, it is a matter of controversy regarding whether this viral internalization occurs via ACE2 or others.

### 4.2. Releasing Bioactive Molecules to Interfere with SARS-CoV-2 Infection 

In addition to internalization, activated platelets achieve direct antiviral effect by releasing a series of bioactive molecules from externalized α granules, one of which is a platelet-derived chemokine: PF4. It has been confirmed that PF4 can suppress HIV infection [151,152]. Similarly, PF4 may bind to SARS-CoV-2 envelope protein and block the attachment of viral particles to host cells, although this hypothesis warrants further experimental validation. However, recent studies have shown that anti-PF4 antibodies activate platelets and contribute to thrombosis in COVID-19, suggesting a different mechanism of platelet PF4 involved in SARS-CoV-2 infection [153]. In addition to PF4, other platelet chemokines such as CCL3 and CCL5, the antiviral activities of which have been confirmed in HIV or influenza infections, may also influence SARS-CoV-2 infection by perturbating the viral envelope (Figure 2) [53,154]. The indirect antiviral effect of platelets mainly comes from their abilities to orchestrate innate or adaptive immune responses against viral infections, among which the platelet–leukocyte interaction triggers a series of downstream antiviral events, which will be discussed as below.

### 4.3. Modulating Innate Immune Cells 

Neutrophils, the most prevalent leukocyte in human blood circulation, undergo interaction with platelets via P-selectin/PSGL1 axis and form heterotypic aggregates, activating Toll-like receptors to execute anti-infection functions (Figure 1 and Figure 2). Blair and colleagues first revealed that TLR2 expressed in human platelets was capable to initiate inflammatory response against bacterial components [155]. As mentioned above, NET is a DNA-based structure, and its formation is termed NETosis, whereby microbes are captured and eliminated [156,157]. It had been reported that NETosis could be induced by secretion of platelet β-defensin 1 under the mediation of Staphylococcus aureus α toxin [158]. Platelet TLR4, capable of recognizing TLR4 ligands like intravascular LPS, allowed the binding of platelets to neutrophils to form NETs [159]. Based on the above findings, we speculate that during SARS-CoV-2 infection, platelet–neutrophil aggregation may enhance the formation of NETs to exert antiviral effects on SARS-CoV-2, but this speculation still warrants the experimental validation.

However, another possibility is that platelet–neutrophil aggregation is more inclined to induce severe COVID-19 symptoms, such as inflammation, thrombosis, or vascular damage. In addition to neutrophils, platelets can interact with another kind of important innate immune cells, dendritic cells (Figure 2). Platelets can not only induce dendritic cells to present antigens to T cells but also be phagocytosed by DCs, followed by the release of serotonin, which is important for T cell proliferation and differentiation [58,160,161]. To date, natural killer (NK) cell–platelet interaction exhibits the least understood effects on antimicrobial immunity. However, some studies had showed that SARS-CoV-2 infection leads to the maturation and activation of NK cells, as evidenced by the enhanced expression of cytotoxic molecules (e.g., perforin, granzyme A, and granzyme B) and cytokines (IFN-γ and TNF-α), as well as increased number of NK cell-platelet aggregates [162]. These findings provide us with clues to speculate that NK cell–platelet aggregates may participate in innate immune responses against SARS-CoV-2.

### 4.4. Enhancing T-Lymphocyte and B-Lymphocyte Function 

According to a previous study, platelet-depleted mice exhibited weakened humoral and cellular immune responses to adenovirus, which were then rescued via platelet infusion, indicating that platelets play a noticeable role in modulating T cell and B cell functions (Figure 1 and Figure 2) [163]. Platelets mediate the activation of cytolytic and helper T cells via CD11b, GPIIb/IIIa, and CD40L, which in turn strongly enhance platelet aggregation. The expression of CD40L (CD154) in platelets is induced under several stimulatory situations, like TLR7 stimulation, and is competent to promote T cell antiviral immunity [164]. León-Ponte et al. found that serotonin, a bioactive factor released by platelet δ-granules, enhances T cell activation via signaling through its 5-HT_7_ receptor [164]. Serotonin also acts as a driver in enhancing T cell proliferation [165]. Compared to platelet-T cell interaction, it is less common for platelets to form aggregates with B cells due to the lack of P-selectin glycoprotein ligand-1 expression on B cell surface [166]. However, an in vitro study has uncovered that human B cells, when coculturing with platelets, increased the class switching recombination and production of immunoglobulin (IgG1, IgG2, and IgG3) via CD40L–CD40 signaling [167]. Platelets generate the majority of circulating CD40L, contributing to the CD40L–CD40 axis that is instrumental for B cell responses [168]. Considering the mentioned facts, it is rational to conjecture that platelets may respond to SARS-CoV-2 infection by enhancing T cell and B cell-mediated immunity.

## 5. Potential Platelet-Targeted Treatment of COVID-19 

Considering the extraordinary thrombotic effects platelets exert during the development of inflammatory conditions, it is promising to utilize antiplatelet drugs or develop novel antiplatelet therapies in controlling or treating COVID-19 [65,169]. A study with a cohort of 7824 consecutive COVID-19 patients revealed that the usage of antiplatelet therapies reduced the mortality risk as well as duration of mechanical ventilation among hospitalized COVID-19 patients without an extra risk of bleeding [170]. Chow et al. and Meizlish et al. both reported that aspirin therapy on hospitalized COVID-19 patients greatly reduced intensive care unit admission, the need for mechanical ventilation, and the incidence of death [171,172]. In addition, two ongoing clinical trials [RECOVERY (NCT04381936) and ACTIV-4 (NCT04505774)] focus on the outcome of platelet P2Y12 antagonist treatment in COVID-19 patients. Among this group of antiplatelet drugs, ticagrelor was reported to significantly reduce thrombo-inflammatory markers in pneumonia patients, including platelet–leukocyte interactions, release of neutrophil extracellular traps, and IL-6 levels, all of which played pathological roles in COVID-19 [173]. In conclusion, antiplatelet therapies are viewed as a promising approach to control the disease progression and even decrease the mortality rate by alleviating the severity of thrombo-inflammatory complications of COVID-19 [174].

In addition to antiplatelet strategies, anticoagulants have been used to ameliorate thrombotic events in COVID-19 [175]. Furthermore, researchers also targeted pathological inflammation to treat COVID-19. Steroids, widely used drugs that relieve inflammatory states by antagonizing the marked pro-inflammatory cytokines or compounds such as NF-κB, have been discussed as a potential supportive treatment for COVID-19 patients, although the side effects of steroids have been reported in managing COVID-19 cases [176,177]. Earlier studies showed that tocilizumab–dexamethasone combination treatments exhibited better protective effects in critically ill patients with COVID-19 compared to the dexamethasone group, indicating tocilizumab—an interleukin-6 receptor antagonist—was a promising drug for COVID-19 [178]. In this respect, researchers explain that the potential effects of tocilizumab in ameliorating coagulation and thrombotic conditions account for the better curative effect of the tocilizumab–dexamethasone combination. This hypothetical mechanism has been supported by evidence that COVID-19 cases taking tocilizumab exhibited both decreased level of D-dimer and reduced incidence of thromboembolic complications [179,180].

## 6. Conclusions

Throughout the course of the COVID-19 pandemic over the past three years, a multitude of studies had reported increased thrombotic events and platelet alterations, notably in terms of platelet count and volume. The escalated platelet activation and aggregation induced by inflammation and endothelial dysfunction in COVID-19 indicate that SARS-CoV-2 coronavirus may affect the function of platelets by either directly invading them or indirectly altering their transcriptome. In accordance with the previous literature, platelet–microbial pathogen interactions are bidirectional and are involved in the pathology of many diseases, which provides insights into about the mechanisms of anti-SARS-CoV-2 activity of platelets. Inspired by the knowledge of interactions between platelets and known pathogens and the anti-microbial activity of activated platelets, we proposed possible mechanisms by which platelets antagonize SARS-CoV-2 coronavirus, including directly killing viruses and recruiting immune cells to eliminate viruses. We also cast eyes on well-developed therapies or ongoing clinical trial of novel drugs targeting platelets and platelet-mediated thrombo-inflammatory complications, which have been reported to exhibit promising therapeutic effects in COVID-19 patients. Overall, we maintain that understanding platelet–SARS-CoV-2 interactions could shed light on how severe COVID-19 complications arise and that modulation of this interaction could lead to new therapeutic possibilities.

## Data Availability

All relevant materials are available from the authors.

## Abbreviations

ACE2	Angiotensin-converting enzyme 2
ADAMTS13	Enzyme A disintegrin and metalloprotease with thrombospondin motifs-13
CMV	Cytomegalovirus
COVID-19	Coronavirus disease 2019
cPLA_2_	Cytosolic phospholipase A2
DVT	Deep vein thrombosis
HSV-1	Herpes simplex virus type 1
ICU	Intensive care unit
IL	Interleukin
MAPK	Mitogen-activated protein kinase
MERS-CoV	Middle East respiratory syndrome coronavirus
NET	Neutrophil extracellular trap
NK cell	Natural killer cell
PF4	Platelet factor 4
PI3K	Phosphoinositide 3-kinase
PRR	Pathogen-recognition receptor
PSGL-1	P-selectin glycoprotein ligand 1
SARS-CoV-2	Severe acute respiratory syndrome coronavirus 2
TLR	Toll-like receptor
TNF-α	Tumor necrosis factor alpha
VEGF	Vascular endothelial growth factor
vWF	von Willebrand factor
